# The Grave of Hope

**Published:** 1880-09

**Authors:** 


### THE GRAVE OF HOPE

Is any large city, and emphatically in this respect, is New York the city of the dead. Not a day, scarce an hour passes, which does not witness some fond anticipation wilted in the grasping; some long cherished expectation withered in an hour; and in an hour the plans of life wrecked for ever.

On a beautiful afternoon of the earliest spring time, we walked Broadway from Trinity church to Union Square, first for months. It occurred to us to step in and see a familiar face, which we had not met since summer. Then, there was the portly mien, the manly eye, the confident expression.—There was the ruddiness of cheek and solidity of muscle which told of vigorous health. In his well-ordered establishment, the piles of goods, the crowd of customers, the activity of clerks, silent, staid, respectful, all spoke of thrift. But now the name was gone, the sign was down. Knowing the house and neighborhood and number, we entered; and new arrangements, and strange faces were presented. Is Mr. —— in?—No! Where is he? Gone! When will he return? Can't tell. The story was soon told. He had failed. Money gone, friends fled, hope prostrate, he had left the city. Whither, no one knew. What to do no one cared. If to return ever, none thought to inquire. What anxieties, what apprehensions, what struggles, what tortures tore that man's heart in the daily battle to keep up his name, which always precedes an honorable failure; and at home what silent meals, what troubled looks, what unaccustomed coldness to children, what unusual and short and almost cross replies to the enquiries of an apprehensive and affectionate wife; what listless meals; what startling dreams; what sleepless nights; what clouds, the clearest morning sun but darkened; what pangs the careless prattle of children struck home upon a loving father's heart, whose thoughts could dwell on nothing but coming demands, dishonored bills and protested notes, we shall never know, but a monument, the grave of hopes of early, vigorous manhood, we have seen.

A dozen blocks on, and within the hour, we thought of another name and looked for it on the street, but looked in vain. It was not be found. Six months earlier, a little old man, of rugged health, of sprightly eye, of active gait, benignant look and joyous good nature, made our acquaintance. At the first visit, he spread out a life cf alternate hopes and fears, of success and failure. But never discouraged, never cast down, he had struggled through his last battle with perplexities and obstacles and delays, which well might have vanquished any heart, and now, believed himself near the topmost round, the battle of half a century almost ended, the victory almost won. A month later, a cloud had come, disappointments muttering thunders sounded in the distance, next the crash, hope died out for ever, and we shall see him no more.

Within the same week, we met a gentleman whom we had not seen for months; then his "transactions" were by the tens of thousands. Every thing prospered with him. He was also a scholar, refined in feeling, elevated in sentiment, having a most generous and manly heart. But to-day he had changed. There was the easy courtesy of the gentleman, but there was at the same time that subduedness of demeanour, which reverses always "impress on the sensitive." I am not worth a shilling to-day, Doctor. I could have put out a hundred and twenty thousand dollars on bond and mortgage, but I wanted "a little more," and I have parted with my last shilling literally. "You are yet young" said we, "look upward and ahead, only those who have failed can *know* that they are men, that knowledge you have, and it is equal to another fortune you did'nt think of."

A very few hours later, we met another, respected and honored at home; he visited foreign lands and stood in foreign courts; assembled thousands had waited on his lips; his utterances were printed in both hemispheres. The morning sun of his life had risen in splendor. His estates were measured by miles. Great men, traveled men, rich men felt complimented to seat him at their tables. But within six months, a change had come over him too. The foretime ready smile came not at his greeting. Furrows had been plowed, and settled sadness had made its deep imprint on that lang syne sunny face. "Money" gone, children scattered, wife worse than dead, was the short, sad history, of budding prospects blighted in an hour, of early flowers wilted in the blowing, of human hopes all perished in a night.

Little know the plain plodding workers of the country, whose farms are paid for, and clear of liens, how happy and stable they should feel, and what little ground there is for the envious thoughts which often rise within them when they hear of men in cities handling money or merchandize by the millions every year, and who in a season spend more than would buy a good sized farm. Four out of five of them die poor, unless they have secreted money from their creditors. The owner of a dozen acres, or a hundred, or a thousand, can go to bed any night and feel secure that he shall wake up next morning with a home of his own, and a living within it, for him and his. On the contrary, the city merchant or banker has no such security. The mail of any morning may bring to him the death warrant of all earthly hopes, and he go home that night the penniless occupant of a brown stone mansion; wife, children and friends all unconsoious of the dreadful change.

Said a merchant the other morning, "your article on life assurance has come a day too late, for I had just taken out a policy for fifteen thousand dollars." On expressing some surprise that a man of his wealth should ever think of his family coming to need so small a sum, he said—"a man in our business can't tell how long he will be worth any thing." And this is precisely the uncertainty which consumes the life of business men in great cities, before half the allotted term has passed away. They take no [illegible] or rest or sleep.

